# A Multi-Objective Task Scheduling Strategy for Intelligent Production Line Based on Cloud-Fog Computing

**DOI:** 10.3390/s22041555

**Published:** 2022-02-17

**Authors:** Zhenyu Yin, Fulong Xu, Yue Li, Chao Fan, Feiqing Zhang, Guangjie Han, Yuanguo Bi

**Affiliations:** 1 School of Computer Science and Technology, University of Chinese Academy of Sciences, Beijing 100049, China; xufulong16@mails.ucas.ac.cn (F.X.); liyue161@mails.ucas.ac.cn (Y.L.); fanchao18@mails.ucas.ac.cn (C.F.); zhangfeiqing17@mails.ucas.ac.cn (F.Z.); 2 Shenyang Institute of Computing Technology, Chinese Academy of Sciences, Shenyang 110168, China; 3 Liaoning Key Laboratory of Domestic Industrial Control Platform Technology on Basic Hardware and Software, Shenyang 110168, China; 4 College of Internet of Things Engineering, Hohai University, Changzhou 213022, China; hanguangjie@gmail.com; 5 Changzhou Key Laboratory of Internet of Things Technology for Intelligent River and Lake, Changzhou 213022, China; 6 School of Computer Science and Engineering, Northeastern University, Shenyang 110167, China; biyuanguo@mail.neu.edu.cn; 7 Engineering Research Center of Security Technology of Complex Network System, Ministry of Education, Shenyang 110167, China

**Keywords:** industrial internet of things, intelligent production line, cloud-fog computing, task scheduling, hybrid heuristics

## Abstract

With the widespread use of industrial Internet technology in intelligent production lines, the number of task requests generated by smart terminals is growing exponentially. Achieving rapid response to these massive tasks becomes crucial. In this paper we focus on the multi-objective task scheduling problem of intelligent production lines and propose a task scheduling strategy based on task priority. First, we set up a cloud-fog computing architecture for intelligent production lines and built the multi-objective function for task scheduling, which minimizes the service delay and energy consumption of the tasks. In addition, the improved hybrid monarch butterfly optimization and improved ant colony optimization algorithm (HMA) are used to search for the optimal task scheduling scheme. Finally, HMA is evaluated by rigorous simulation experiments, showing that HMA outperformed other algorithms in terms of task completion rate. When the number of nodes exceeds 10, the completion rate of all tasks is greater than 90%, which well meets the real-time requirements of the corresponding tasks in the intelligent production lines. In addition, the algorithm outperforms other algorithms in terms of maximum completion rate and power consumption.

## 1. Introduction

With the development of information and communication technologies, such as wireless sensor networks [1], industrial Internet of Things (IIoT) [2,3], and cloud computing [4], the transformation and upgrading of manufacturing technology has been promoted, which makes traditional manufacturing shift to intelligent manufacturing. The production equipment, transmission devices, sensors, and other terminal devices in the intelligent production lines have been connected based on various heterogeneous communication networks, making the traditional information islands evolve to the interconnection model. Meanwhile, the extensive use of intelligent devices has generated large amounts of tasks requiring real-time processing [5]. Cloud computing was initially considered the primary enabler capable of processing the massive data generated by IIoT devices. However, there are many problems in cloud computing, the most prominent of which are mainly manifested in the following two aspects: transferring large-scale data from the IIoT devices to the cloud may not be effective, and in some cases, it may not even be feasible due to bandwidth limitations. On the other hand, the more considerable geographic distance between the intelligent edge device and the cloud service center may lead to higher service delays [6], which violates the quality of service requirements for customer requests, such as ultra-low latency requests in intelligent production lines.

A computing paradigm closer to connected devices is needed to solve the problems mentioned above. Fog computing/edge computing, an extension and improvement of cloud computing, deploys fog nodes with certain computing and storage capabilities near terminal devices, enabling cloud services to migrate to the edge of the network for faster response to requests for time-sensitive tasks [7]. However, fog computing cannot completely replace cloud computing. In contrast, both technologies can work together to improve latency and reliability, reduce response time, and are widely used in many fields [8]. For example, in [9], one strategy based on cloud-fog computing was proposed for the virtual reality system of the Industry 4.0 shipyard.

There are some urgent problems in cloud-fog computing and intelligent production line task scheduling. There are considerable differences in computing power, storage, and communication capabilities among various fog nodes in a cloud-fog computing environment. The tasks generated by terminal devices are highly heterogeneous in real time and energy consumption [10,11]. In the intelligent production line, different task service sequences bring different delays. In particular, some time-sensitive tasks, such as production line early warning and high delays caused by unreasonable task scheduling strategies, can result in catastrophic results.

On the other hand, in intelligent production lines where batteries power many fog nodes, different task scheduling strategies lead to different energy consumption, which inevitably brings many problems. For example, a study found that when a device cannot be charged in time, frequent data exchange, transmission, and processing can cause the battery’s life to be significantly reduced due to instantaneous discharge, thereby causing a data leakage security risk [12]. It is a tremendous challenge for intelligent production lines to ensure low delay to complete tasks and effectively reduce the power consumption of fog nodes. However, there are still few studies on task scheduling for intelligent production lines in our literature survey. In the intelligent production line, we take latency and energy minimization as the optimization direction for task scheduling, considering the time-sensitive differences of various types of tasks and heterogeneity of the running power consumption of different computing nodes.

The task scheduling problem is a challenging non-deterministic polynomial difficulty (NP-hard) problem [13]. To date, the hybrid heuristic algorithm can combine the advantages of various heuristic algorithms to solve the task scheduling problem with high accuracy [14]. Therefore, we use a hybrid heuristic algorithm to solve the proposed optimization problem, realize the efficient use of cloud computing resources, and reduce the overall consumption of computing resources while satisfying low latency. The main contributions of our study are listed as follows:

1. We present a multi-objective task scheduling optimization problem in intelligent production lines. A multi-priority task scheduling strategy based on a cloud-fog computing architecture is used to solve this problem, achieving a fast response to intelligent production line tasks and reducing energy consumption.
2. A new task scheduling algorithm hybridizing the MBO and ACO is implemented in our study. The improved MBO and ACO more easily converge. More importantly, this is the first time that MBO has been applied to task scheduling scenarios in intelligent production lines.
3. We establish an intelligent production line simulation experiment platform based on C++ and evaluate the proposed algorithm. The results show that it is superior to other strategies in terms of average delay and power consumption.

The remainder of the paper is organized as follows. In Section 2, we describe the related work. In Section 3, we introduce the system model and problem formulation. In Section 4, we propose a task scheduling algorithm. Section 5 discusses the performance evaluation. Finally, in Section 6, we give a brief conclusion.

## 2. Related Work

In recent years, with the continuous development of fog computing and the requirements of terminal equipment for real-time performance and energy consumption, cloud-fog computing has become a trend, and task scheduling under cloud-fog computing has become a necessary research hotspot. We reviewed many studies on task scheduling for cloud and fog computing and listed them below.

### 2.1. Cloud Computing Task Scheduling

Cloud computing provides rich computing, storage, and other application services for industrial production, bringing huge energy consumption. With increasing attention being paid to carbon neutrality [15], it is imperative to improve the task allocation efficiency of cloud computing and reduce energy consumption in the industry. To obtain the best performance of task scheduling in cloud computing, Rajakumari et al. [16] proposed a fuzzy hybrid particle swarm parallel ant colony algorithm. This algorithm improved task scheduling with the objectives of minimizing execution and waiting time, increasing system throughput, and maximizing resource utilization. However, the study did not consider energy efficiency. Under the premise of ensuring cloud computing service quality, Rao et al. [17] completed the coordination and energy consumption minimization of data center scheduling. Lin et al. [18] proposed two IoT-Aware multi-resource task scheduling algorithms to reorder tasks based on priority, and task scheduling using heuristic algorithms. The simulation results showed that this method could reduce energy consumption as much as possible while ensuring the response time and load balancing results of IoT tasks. Although the above two studies guide task allocation, the difference in power consumption of the computing unit itself was not considered for task scheduling.

### 2.2. Fog Computing Task Scheduling

Fog nodes have differences in distribution and computing capacity. Effectively scheduling tasks requested by terminal devices can reduce service delay and energy consumption [19]. In the field of intelligent manufacturing, Mithun et al. [20] proposed a solution to the fog computing task offloading problem. This solution modelled the optimization problem mathematically and used quadratic constraint quadratic programming to solve the de-weighting problem, and finally solved the optimization problem by the semi-deterministic relaxation method. Chekired et al. [21] proposed a self-adaptive fog computing multi-objective optimization task scheduling method, which solved the multi-objective optimization problem of fog computing task scheduling with the total execution time and resource cost of tasks as the optimization objectives. Both studies provided excellent ideas for reducing task processing and waiting time, but neither reduced the task processing power consumption. In the research of Hang et al. [22], a joint computing offloading and wireless resource allocation algorithm based on Lyapunov optimization was proposed to minimize system delay, energy consumption, MDs weighting, and other associated costs. However, the study ignored the interaction between the cloud center and fog nodes and only divided the main problem into several sub-problems in each time slot and then allocated them to different fog nodes for calculation. Suppose we encounter a task that requires a large number of computing resources and is not divided, such as intelligent production line image processing. In that case, the resource-constrained fog node cannot process it, resulting in the task being unable to be completed. For the task offloading problem in fog computing, Keshavarznejad et al. [23] proposed a multi-objective optimization problem of energy consumption and delay, which was solved using a hybrid heuristic algorithm. The results showed that the best trade-off was obtained between the probability of offloading and the energy consumption required for data transmission. Regrettably, this approach did not categorize tasks to respond to urgent tasks quickly.

### 2.3. Cloud-Fog Computing Environment Task Scheduling

In cloud-fog computing, scheduling IoT tasks to reduce the delay and energy of time-sensitive tasks has attracted the attention of researchers [24]. Abdelmoneem et al. [25] proposed a mobile-aware task scheduling and allocation method under the cloud-fog computing paradigm, which greatly reduced the energy consumption of task processing and task delay. This method effectively solved the task assignment of the sensing device in the mobile scene. However, it was unsuitable for intelligent production lines, etc., where the sensors were mostly fixed. Mokney et al. [26] studied IoT tasks with dependencies under cloud-fog computing, proposed modeling workflow planning as a multi-objective optimization problem, and designed a compromise solution regarding response time, cost, and maximum completion time. The proposed algorithm was superior in solving the scheduling problem that depended on task flow. However, it did not solve the scheduling problem of independent tasks. The algorithm obtained the Pareto optimal solution, which could not meet the urgent tasks that require ultra-low time response. Bisht et al. [27] studied the problem of rapid task response in the cloud-fog computing environment. They proposed a workflow scheduling method with the smallest maximum completion time and energy consumption. The research was based on task length for scheduling, and could not respond quickly to urgent and complex tasks.

Based on the above studies, we find that the task scheduling problem in the cloud-fog computing environment is a research hotspot in the IoT field, and the existing research cannot meet the requirements of low latency and low power consumption for multi-priority task scheduling in intelligent production lines.

### 2.4. Heuristic Algorithm to Solve the Task Scheduling Problem

Heuristic algorithms are a subset of the artificial intelligence field, which is popular in solving different optimization problems and is often used to solve task scheduling problems [28,29]. Common heuristic algorithms include ant colony optimization (ACO) [30], genetic algorithm (GA) [31], particle swarm optimization (PSO) [32], simulated annealing algorithm (SAA) [33], Grey Wolf Optimizer (GWO) [34], monarch butterfly optimization algorithm (MBO) [35], and so on. The MBO, with simple computational procedures and fewer parameters, is more easily to implement in these algorithms. MBO is suitable for solving small-scale search problems and is widely used in many fields [36]. The ACO can search on a large scale. To improve its search process, it can have excellent exploration and development capabilities at the stage of generating the optimal solution [28].

Many scholars have proposed that multiple single heuristics should be combined into a hybrid algorithm to obtain better task scheduling performance. In [37], a meta-heuristic-based service allocation framework was designed to schedule edge service requests using three meta-heuristic techniques, PSO, binary PSO, and Bat algorithm (Bat). The experimental results showed that the framework solved the dual objective minimization problem of energy consumption and maximum completion time. Ref et al. [38] proposed a hybrid bionic algorithm for cloud computing task scheduling and resource management. Fu et al. [39] improved the service quality of cloud computing by adopting the task scheduling optimization algorithm of hybrid PSO and GA. There are many documents on cloud computing task scheduling algorithms, but few studies on hybrid heuristic scheduling algorithms for task scheduling under cloud-fog computing.

Inspired by the above studies, we use a hybrid heuristic algorithm of MBO and ACO to solve the intelligent production line task scheduling problem.

## 3. System Model and Problem Formulation

In this section, we present the mathematical description of the task scheduling problem for intelligent production lines under cloud-fog computing.

### 3.1. System Architecture

**Figure 1:**
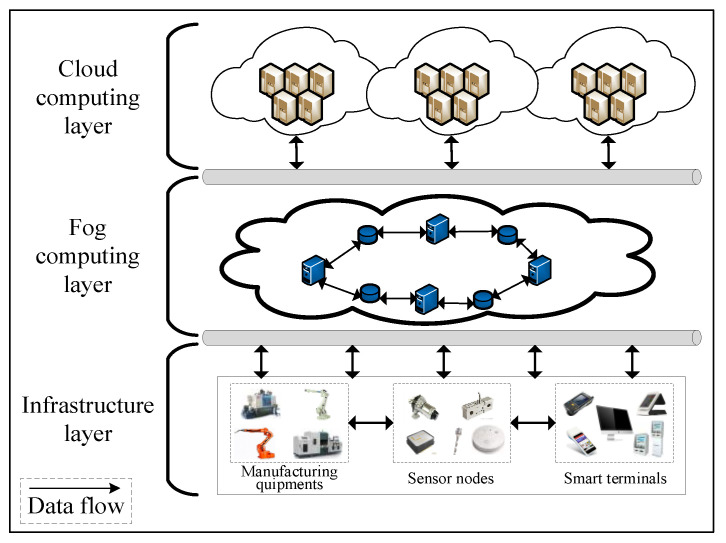
The architecture of intelligent production line system architecture based on cloud-fog computing.

We built a cloud-fog computing architecture to solve the increasing problems of delay-sensitive and computationally intensive tasks in intelligent production lines. The system architecture is given in Figure 1; it consists of three layers: infrastructure layer, fog computing layer, and cloud computing layer.

Infrastructure layer: The infrastructure layer consists of terminal devices with different functions, such as various sensors, processing devices and various smart terminals. Smart terminals handle simple tasks locally but are unable to perform complex tasks in real time.

Fog computing layer: The fog computing layer is mainly composed of fog nodes. These are servers with certain computing, communication, and storage capabilities in intelligent production lines, such as smart sensors, smart processing devices, and intelligent multimedia devices. This layer can sense the requests of intelligent production line terminals and provide various services in real time, which can greatly reduce the delay of task processing and ensure the quality of service of real-time applications.

Cloud computing layer: The cloud computing layer consists of clusters with huge computing and storage capacity, providing remote services for intelligent production lines to handle complex computing tasks.

### 3.2. System Model

#### 3.2.1. Description of System Model

**Figure 2:**
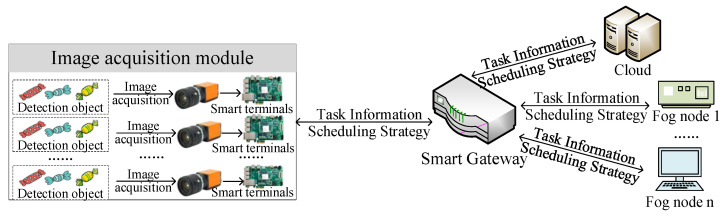
The image processing process of intelligent production lines.

In fields that require high real-time task processing, such as intelligent production lines and smart hospitals, ensuring the task processing reliability has always been a crucial issue [40]. It is necessary to analyze and process various data tasks in real time, such as material information reading and multi-axis robot posture analysis, when the intelligent production line is running. For example, the intelligent production line for personalized production of candy packaging realizes any combination of different shapes, colors and quantities of candy packaging. The machine vision-based candy sorting system is the key to complete the candy packaging, and the data uploaded by its image acquisition module need to be analyzed and processed in real time during the operation. Figure 2 depicts the flow of image processing.

**Figure 3:**
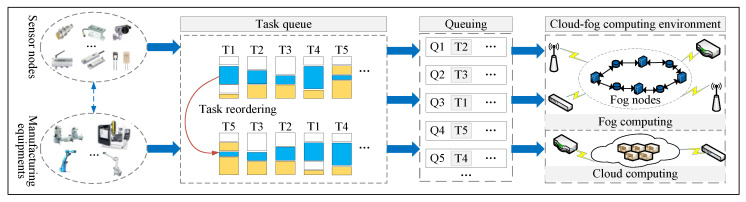
Task processing flow in the cloud-fog computing environment.

However, the processing and analysis capabilities of fog nodes are limited. With the increasing number of tasks to be processed, an unreasonable task allocation mechanism often leads to an increase in task delay, a decrease in completion and an increase in energy consumption. Consequently, we propose a task scheduling algorithm based on cloud-fog computing. The proposed algorithm uses a hybrid heuristic scheduling algorithm to reasonably allocate tasks to fog nodes and cloud servers, and thus solves the above problems. The tasks generated by the terminals of the intelligent production line are processed in the cloud-fog computing environment as shown in Figure 3. We classify the tasks generated by the terminal device according to their urgency and sort them according to their priority. The scheduling algorithm distributes the sorted tasks to the fog nodes and cloud servers to ensure that the tasks will be served as much as possible.

#### 3.2.2. Latency Model and Energy Consumption Mode

First, we assume that there are k tasks $\;T=\left\{T_{1},\;T_{2},\;\ldots ,\;T_{k}\right\}$, the priority of each task is $PList=\left\{\lambda_{1},\lambda_{2},\;\ldots ,\;\lambda_{k}\right\}$, and they are independent of each other. Among them, each task $T_{i}$ can be expressed as a three tuple $T_{i}=\left\{D_{in}\left(i\right),Y\left(i\right),D_{out}\left(i\right)\right\}$. $D_{in}\left(i\right)$ represents the input data volume of the task, in kbit, Y(i) defines the load of the task, in MIPS, and $D_{out}\left(i\right)$ indicates the output of the task, in kbit. Denote n as the number of service nodes $F=\left\{F_{1},F_{2},\;\ldots ,\;F_{n}\right\}$, which includes fog nodes and a cloud service. For the convenience of numbering, we let $F_{n}$ denote the cloud service. We define a non-negative integer variable $X_{i,j}$ to indicate whether the node i can handle service request number j, such as:

(1) $$X_{i,j}=\{\begin{array}{c} 1,\;\;if\;node\;i\;handle\;task\;j\\ 0,\;\;others \end{array}.$$

Since fog nodes have heterogeneous resources, they provide different types of computing services for terminal devices. To allow each task to be served, we ensure that at least one node can serve each task when setting up the nodes. Formally, we have:

(2) $$\underset{j\in T}{\sum }X_{i,j}\geq 1\;\;\forall \;i\;\;\in \;F.$$

Data transmission in the task from the terminal device to the node is often limited by the transmission speed, and the transmission rate is usually defined as follows:

(3) $$Trans_{i}=C\times \log_{2}\left(1+\frac{p_{i\times }H}{N_{0}+H\times \sum_{j\in I,j\neq i}p_{j}}\right),\;\forall i\in T$$

where $p_{i}$ is the transmission power of the fog node, $N_{0}$ is the Gaussian noise power in the channel, C is the bandwidth provided by the access network, and H is the channel gain parameter between the fog nodes. In this study, we assume that the information gain parameters between all fog nodes are the same. In addition, it can be seen from Equation (3) that when a channel is used at the same time, the data transmission rate will be reduced.

Then, the total transmission time of the node $F_{i}$ receiving the task and sending the task after processing is expressed as:

(4) $$Time_{i}^{trans}=\sum_{j}^{k}X_{i,j}\times \left(\left(D_{in}\left(j\right)+D_{out}\left(j\right)\right)/Trans_{i}\right),\forall i\in F.$$

We use $f=\left\{f_{1},f_{2},\;\ldots ,\;f_{n}\right\}$ to define the ability of each node to process tasks, and the unit is MPIS. The time to complete a process in the node should be the time it is served, so we can obtain this formula:

(5) $$Time_{i}^{deal}=\sum_{j}^{k}X_{i,j}\times \left(Y\left(j\right)/\left(f_{i}\right)\right),\forall i\in F$$

Since all tasks are served in the allocated order, the subsequent tasks in each node must wait for the previous processing to be completed before being served. Therefore, the queuing time of task j in each node i is:

(6) $$Time_{i,j}^{queue}=\sum_{j=1}^{t-1}X_{i,j}\times Time_{i}^{deal},\;X_{i,t}=1,\forall i\in F,\forall t\in T.$$

Then, the total queue time of each node task is:

(7) $$Time_{i}^{queue}=\sum_{j=1}^{k}X_{i,j}\times Time_{i,j}^{queue},\forall i\in F.$$

Obviously, we can find from Equations (4)–(7) that the total time for each node to complete all scheduled tasks can be expressed as:

(8) $$Time_{i}^{total}=Time_{i}^{trans}+Time_{i}^{deal}+Time_{i}^{queue},\forall i\in F.$$

It should be noted here that since the work of each node is independent of each other, the time for all tasks to complete is equivalent to the longest time the task takes. The maximum delay can be expressed as:

(9) $$Time_{max}=argmax_{i\in I}\left(Time_{i}^{total}\right)$$

In this study, we use $p^{trans}=\left\{p_{1}^{trans},\;p_{2}^{trans},\;\ldots ,\;p_{n}^{trans}\right\}$ to represent the transmission power of the node. Combined with Equation (4), we can obtain the transmission energy consumption of each node as:

(10) $$E_{i}^{trans}=Time_{i}^{trans}\times p_{i}^{trans},\forall i\in F.$$

As above, $p^{deal}=\left\{p_{1}^{deal},\;p_{2}^{deal},\;\ldots ,\;p_{n}^{deal}\right\}$ represents the service power of node i. Taking into account Equation (5), the energy consumption of each node when providing services is:

(11) $$E_{i}^{deal}=Time_{i}^{deal}\times p_{i}^{deal},\forall i\in F.$$

The power consumption generated by the task in queuing is extremely small, and we do not calculate the energy consumption when the task is waiting. Then, the total energy consumption of each node is:

(12) $$E_{i}^{total}=E_{i}^{deal}+E_{i}^{trans},\forall i\in F.$$

From Equations (10)–(12), we can easily know that the total energy consumption generated by nodes in the entire task scheduling cycle can be expressed as:

(13) $$E_{total}=\sum_{i}^{n}E_{i}^{total},\forall i\in T.$$

#### 3.2.3. Time Delay and Power Consumption Evaluation Model Based on Task Priority

In intelligent production lines, tasks with different degrees of urgency have different response time requirements, yet usually the time tolerance for urgent tasks is low. To ensure that urgent tasks are processed quickly, we classify the tasks in the buffer list according to their time tolerance. We simply classify the tasks into two categories: the tasks with lower time delay requirements are considered high-priority tasks, and the others are considered low-priority tasks. The priority of a task is denoted by λ,

(14) $$\lambda =\{\begin{array}{c} 1,\;\;high-priority\;tasks\\ 2,\;\;lower-priority\;tasks \end{array}$$

The delay and power consumption of different nodes in the fog computing layer differ when processing the same task. When using heuristics to search for potentially good solutions, the search direction should be adaptively adjusted according to the priority of the task. For high-priority tasks, lower service latency should be the main search direction, while for low-priority tasks with lower latency requirements, lower power consumption should be the search target. Therefore, we construct a latency-power evaluation model based on the different requirements of latency and power consumption for the two priority tasks using the properties of the exponential function.

The evaluation formula is:

(15) $$\Gamma =\frac{1}{\vartheta \times e^{Time_{max}\times \left(2-\lambda \right)}+\mu \times e^{E_{total}\times \left(\lambda -1\right)}}$$

From the formula, we can find that when it is a high-priority task, the value of the evaluation function is affected by the time delay. In the opposite case, it is affected by energy consumption parameters. Among them, ϑ and μ represent the coefficients of latency and energy consumption, respectively.

Through the description of the above formula, our goal formula becomes clear, including the completion time of all tasks and the energy consumption of all nodes. Our goal is to ensure that all are completed on time while minimizing energy consumption, namely:

(16) Minimize {Γ}.

$$s.t.\;\left(c1\right):\underset{j\in T}{\sum }X_{i,j}\geq 1\;\;\forall \;i\;\in \;F,$$

$$\left(c2\right):\underset{i\in F}{\sum }X_{i,j}\geq 1\;\;\forall \;j\;\in \;T,$$

$$\left(c3\right):\underset{i\in F}{\sum }\underset{j\in T}{\sum }que_{i,j}=1,$$

$$\left(c4\right):Time_{max}\;\leq MaxTime,$$

$$\left(c5\right):E_{i}^{total}\;\leq MaxPower,\;\forall \;i\;\;\in \;F.$$

where we use constraints (c1) and (c2) to ensure that each task can be served, and each node will provide at least one service. In (c3), $que_{i,j}$ represents the correspondence between node i and task j after scheduling, and we constrain each task to be served by only one node. (c4) and (c5) constrain the latency and energy consumption, respectively.

## 4. Task Scheduling Algorithm Design

The task scheduling problem in cloud-fog computing has difficulty obtaining the optimal solution in polynomial time due to the many variables and constraints in the objective function [41]. To minimize the delay of all tasks and reduce the energy consumption of nodes, we combined the advantages of the MBO and ACO to design a hybrid heuristic algorithm. It solves the optimal task scheduling problem in the cloud-fog computing environment.

### 4.1. Task Rescheduling Strategy

**Figure 4:**
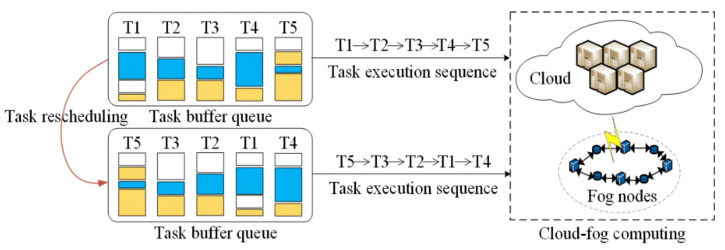
Rescheduling process based on the order of task priority.

The traditional task scheduling sequence usually adopts the first-come, first-served method, which is suitable for tasks with the same priority. However, this is generally not feasible for time-sensitive tasks because the sequential service will cause time-sensitive tasks to be ranked after non-sensitive jobs. We use a merge sorting algorithm to prioritize the tasks to avoid this problem. The sorted sequence ensures that the high-priority tasks can be served first. We take Figure 4 as an example. The blue stripes denote the priority of each task, and the service order of their initial jobs is T1→T2→T3→T4→T5. After the task rescheduling strategy is adjusted, the order assigned to the cloud before computing is changed to T5→T3→T2→T1→T4.

### 4.2. Monarch Butterfly Optimization

The MBO algorithm [35] is a novel population intelligence algorithm that simulates the migration process of monarch butterflies in nature. The MBO algorithm is often used to solve some optimal solution problems with small solution spaces [42]. Based on these studies, we improve the MBO algorithm to address small-scale task scheduling problems to improve efficiency. The implementation process of the improved monarch butterfly is shown in Algorithm 1.
**Algorithm 1:** The Improved Monarch Butterfly Optimization**Input:** 
Task queue, Node list Τ,  Ν,  M
**Output:** The optimal path 1:  **Initialize:**
P,  M1,  M2,  peri,  p,  BAR,  ϑ,  μ 2:  Reorder task priority using merge sorting method to get queue Ψ. 3:  **for** mb = 1; mb <= M; mb++ **do** 4:    Set an initial value for each monarch butterfly. 5:  **end** 6:  **for**
*t* = 1; *t* <= Τ; *t*++ **do** 7:    According to Equation (25) get the task assignment sequence. 8:    Evaluate the fitness value of each individual according to Equation (14). 9:    Sort according to the fitness value of the individual.10:    Select the optimal path.11:    Save the two monarch butterflies with the best fitness values.12:    **for** mb = 1; mb <= M1; mb++ **do**13:      Use the DMMO to update SP1.14:    **end**15:    **for** mb = 1 + M1; mb <= M1+M2; mb++ **do**16:      Use the BAO to update SP2.17:    **end**18:    Combine SP1 and SP2 to generate a new population.19:    Use the two elites to replace the worst two.20:  **end**

#### 4.2.1. Differential Mutation Transfer Operator

The differential evolution algorithm [43] was proposed by American scholars Storn and Price in 1995. It uses mutation, crossover, and selection operations to simulate gene mutation behavior during biological evolution. Generally, heuristic algorithms searching near the better solution can have a greater chance of finding the optimal global solution. Inspired by this, we propose replacing the original migration operator of MBO with a differential mutation migration operator. Using this strategy, we can perform differential evolution mutation operations on the better individuals in the population to achieve the search near the better solution. The differential mutation migration operator (DMMO) can be expressed as:

(17) $$s_{i,k}^{t+1}=\{\begin{array}{c} s_{r_{1},k}^{t}+\gamma \times \left(s_{best,k}^{t}-s_{r_{1},k}^{t}+\Delta \right),r\leq p\\ s_{r_{2},k}^{t}+\gamma \times \left(s_{best,k}^{t}-s_{r_{2},k}^{t}+\Delta \right),r>p \end{array}$$

where γ=[0,2] represents the coefficient of variation, $s_{best,k}^{t}$ is the position of the best individual in each round of iteration, and Δ represents the difference vector, which is expressed as:

(18) $$\Delta =s_{q_{1},k}^{t}-s_{q_{2},k}^{t}$$

where $\;s_{q_{1},k}^{t},s_{q_{2},k}^{t}$ represent the positions of two monarch butterflies randomly selected, and the constraints are:

(19) $$q_{1}=\left\{1,2,\cdots ,M\right\},\;\;q_{2}=\left\{1,2,\cdots ,M\right\},\;\;q_{1}\neq q_{2}\neq r_{1}\neq r_{2}.$$

From Equation (21), the fitness value of the mutation position and the fitness values corresponding to the other three different parts can be calculated according to the difference to obtain the mutation vector, and the weight of the mutation vector is added to a randomly selected monarch butterfly position. In terms of the fitness value, a mutation fitness value carrying diversified information is generated so that the mutation location accepts the mutation fitness value, increasing the diversity of the population and significantly improving the ability of the algorithm to search globally.

#### 4.2.2. Hybrid Encoding

The MO and BAO operators in the MBO algorithm are similar to the calculation of the continuous problem. However, each feasible solution to the task scheduling problem corresponds to a set of positive integers representing node numbers. Then, it is not possible to directly apply the basic MBO algorithm to solve such problems.

We design a simple and effective hybrid coding mechanism based on the task requirements in cloud-fog computing. We represent each candidate solution as a two-tuple 〈X, Y〉. Among them, $\ln\left(X\right)\in {\left[-a,a\right]}^{n}$ is a real-valued vector, which constitutes the search space. In this paper, the parameter a takes a value of 5.0. Y ∈ [1,N] represents the solution space of the problem and is a vector of positive integers, and *N* denotes the number of nodes. We construct a mapping relationship from continuous space to discrete space:

(20) h(x)=k,when(k−1)/N≤sig(x)<k/N,k∈[1,N]

where $sig\left(x\right)=1/\left(1+e^{-x}\right)$ is the sigmoid function.

Therefore, the candidate solution $S=\left\{s_{1},s_{2},\cdots ,s_{M}\right\}$ can be evaluated by the objective function Γ.

### 4.3. Improved Ant Colony Algorithm

The probabilistic algorithm ACO was designed by Marco Dorigo to find an optimal path, inspired by the behavior of ants in the process of searching for food. The ACO algorithm has many excellent properties and is mainly used to solve task scheduling problems with complex solution set [28,30]. We improved the ACO algorithm to address large-scale task scheduling problems, and it is convenient to obtain the optimal task service strategy faster. The implementation of the improved ACO algorithm is shown in Algorithm 2.
**Algorithm 2:** The Improved Ant Colony Optimization**Input:** 
$Task\;queue,\;Node\;list\;Τ,N_{ant}$
**Output:** The optimal path
 1:  **Initialize:**
$\alpha ,\;\;\beta ,\;\;\rho ,\;\;\varphi_{0},\vartheta ,\;\;\mu$

 2:  Reorder task priority using merge sorting method to get queue Ψ.
 3:  
for t=1; t <= Τ; *t*++ **do**
 4:    
for ant=1; ant <= Ν; ant++ **do**
 5:     Select the first task in the task queue.
 6:     
$\mathrm{Randomly}\;\mathrm{select}\;\mathrm{node}\;j\;\mathrm{to}\;\mathrm{start}\;\mathrm{the}\;\mathrm{first}\;\mathrm{task},\;\mathrm{and}\;X_{1,j}=1$.
 7:    
**end**
 8:    
for ant=1; ant <= Ν; ant++ **do**
 9:     
for i ∈ Ψ **do**10:       
$\mathrm{Select}\;\mathrm{the}\;\mathrm{next}\;\mathrm{node}\;\mathrm{based}\;\mathrm{on}\;X_{i,j}=1.$
11:       Calculate the probability based on Equation (21).12:       Determine the node and record the route.13:       Update local information based on Equation (26).14:     **end**15:     Select the current optimal path.17:    **end**18:    Update global information according to Equation (23).19:    Select the optimal path.20:  **end**

#### 4.3.1. Path Construction

In the ACO algorithm, each artificial ant randomly selects a position as the starting point before leaving and maintains a path memory vector to store the positions that the artificial ant passes in turn. The position here refers to cloud and fog nodes. In each step of constructing the path, the artificial ant chooses the following location to reach according to the rule of random proportion. The random probability is calculated according to the following formula:

(21) $$P_{ij}^{k}\left(t\right)=\{\begin{array}{cc} \frac{{\left[\iota_{ij}\left(t\right)\right]}^{\alpha }\times {\left[\eta_{ij}\left(t\right)\right]}^{\beta }}{\sum_{k\in allowed_{k}}{\left[\iota_{ij}\left(t\right)\right]}^{\alpha }\times {\left[\eta_{ij}\left(t\right)\right]}^{\beta }}, & if\;j\in allowed_{k}\\ 0, & others \end{array}$$

$\mathrm{where}\;P_{ij}^{k}\left(t\right)$ represents the probability that node j needs to provide services when node i provides services at time t. $\iota_{ij}\left(t\right)$ denotes the intensity of the pheromone from node i to j at time t, $\eta_{ij}\left(t\right)$ indicates the visibility from node i to j at time t, $allowed_{k}$ represents the set of nodes that have not been visited, and α and β are two constants, that represent the weighted value of pheromone and visibility, respectively.

$\eta_{ij}\left(t\right)$ is determined by Equation (14):

(22) $$\eta_{ij}\left(t\right)=\frac{1}{\vartheta \times e^{\tau_{ij}\left(t\right)\times \left(2-\lambda \right)}+\mu \times e^{\pi_{ij}\left(t\right)\times \left(\lambda -1\right)}}$$

where $\tau_{ij}\left(t\right)$ represents the time spent from nodes i to j at time t, and $\pi_{ij}\left(t\right)$ represents energy consumption. λ is the priority of the task mentioned in Equation (13), where the multi-objective task scheduling is the denominator of Equation (22), and the larger the value of the fitness function is, the better the solution.

#### 4.3.2. Pheromone Update

The pheromone update method is the key to the colony ant algorithm. A certain amount of pheromone is assigned to each route at the beginning of the algorithm. The pheromone is an incentive that encourages ants to explore close to the optimal solution, and the management of pheromones directly affects the algorithm’s efficiency. If there are too many pheromones in the current best path, the result may not jump out of the local optimum. In contrast, it might cause the algorithm to converge too slowly. In the following, we introduce the pheromone update strategy we adopted, which can obtain the global optimal solution and accelerate the convergence speed of the algorithm.

##### Global Pheromone Update

When each generation of artificial ants completes the iteration, all trajectories will be updated globally as follows:

(23) $$\varphi_{ij}^{k}=\left(1-\rho \right)\times \varphi_{ij}^{k}+\Delta \varphi_{ij}^{k}.$$

where ρ is the proportion of disappearing pheromones, and $\Delta \varphi_{ij}^{k}$ is the newly generated pheromones in the trajectory from node i to j. The generation of a new pheromone depends on whether it is the trajectory of the optimal solution, namely:

(24) $$\Delta \varphi_{ij}^{k}=\{\begin{array}{c} 1/\chi ,\;\;if\;i\to j\;is\;involved\;in\;the\;best\;solution\\ 0,\;\;\;others \end{array}.$$

where χ represents the length of the path.

##### Local Pheromone Update

After each artificial ant chooses a path, the pheromones of the two targets will evaporate in a particular proportion to prevent premature convergence. The local update procedure is as follows:

(25) $$\varphi_{ij}^{k}=\left(1-\rho \right)\times \varphi_{ij}^{k}+\varphi_{0}.$$

where ρ is the evaporation rate of the pheromone set in advance, and $\varphi_{0}$ is the initial pheromone concentration. With the local renewal of pheromones, artificial ants tend to seldom untraveled paths, thereby potentially increasing the diversity of solutions.

### 4.4. Hybrid Heuristic Task Scheduling Algorithm

**Figure 5:**
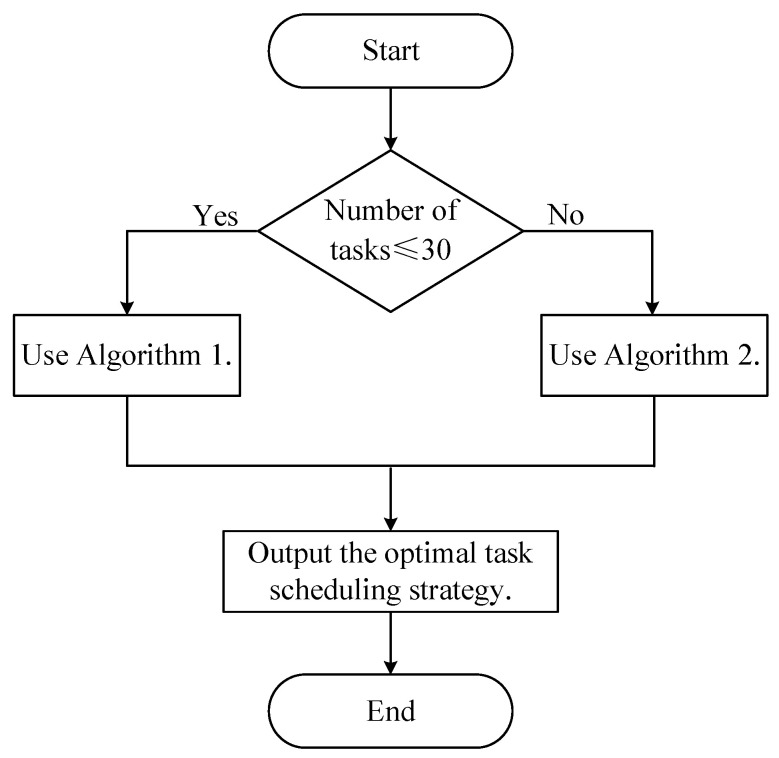
The flowchart of HMA algorithm.

The MBO algorithm and the ACO algorithm are introduced in detail above, and we improved these two algorithms. However, each algorithm has its shortcomings in solving the task scheduling problem. For example, when a large number of tasks must be scheduled, although the search speed of the MBO algorithm is relatively fast, the global search capability becomes weaker, and the search accuracy also decreases. However, for the ACO algorithm, although it has high accuracy, the search speed is low. To obtain critical values for the performance of the two algorithms, we conduct several comparison experiments and find that better scheduling is obtained using the improved MBO algorithm when the number of tasks is less than or equal to 30, while the ACO algorithm performs well in other cases. Based on this, we design a new hybrid MBO–ACO (HMA) algorithm to fully utilize the performance of both algorithms. The threshold is set to 30, and the improved MBO (IMBO) algorithm is used for task scheduling when the number of tasks is less than or equal, and the improved ACO (IACO) algorithm is used when it is greater than. This enables adaptive selection of the optimal scheduling algorithm based on the number of tasks. The flowchart of the HMA algorithm is shown in Figure 5.

#### Time Complexity Analysis

The time complexity of our strategy depends on:

- The time complexity of the task classification strategy.

Task sorting is performed by the merge sort algorithm, so the average time complexity of task sorting is O(n∗logn).

- The time complexity of task scheduling.

The task scheduling strategy is based on MBO and ACO, whose complexity is usually measured in terms of average convergence and is influenced by the number of populations and the number of iterations. However, the randomness and group nature of these algorithms lead to complex and variable stochastic processes, which adds difficulties to the time complexity analysis of the algorithms [44]. We can only approximate the time complexity. The time complexity of the MBO algorithm is approximately O(m×n×m×T), and the ACO is approximately O((n−1)×n×m×T). Here, n represents the number of tasks, m is the number of populations, and T denotes the number of iterations.

## 5. Performance Evaluation

In this section, we perform simulations to verify the feasibility of the proposed method. We present the simulation environment and compare its performance with that of the conventional method. The results further validate the effectiveness of the proposed strategy.

### 5.1. Simulation Settings

The experiment is conducted on a computer with a 3.7 GHz AMD Ryzen 5, 3400 G CPU and 16 G memory storage space. We build a cloud-fog computing architecture and task scheduling model simulation platform using C++ based on a task scheduling scenario in the intelligent production line. To facilitate the performance comparison between algorithms, we set the transmission rate between fog nodes as 3 M/s and that between cloud nodes and fog nodes as 10 M/s. The simulation parameters are shown in Table 1. The parameter settings in Algorithm 1 and Algorithm 2 are shown in Table 2.

### 5.2. Performance Evaluations

To highlight our proposed HMA task scheduling algorithm, we compare two existing scheduling methods: first-come-first-served (FCFS) scheduling [45] and only cloud service methods [18]. We also compare the performance of the IMBO algorithm or the IACO algorithm running alone. Finally, we experiment with a priority-based task reordering strategy. We execute all the algorithms 30 times and average the results to reduce the error due to randomness.

We summarize the results of previous studies and identify three evaluation indicators to evaluate the experimental results. The first is the maximum completion time [37,46,47,48,49], which is the time required to complete the last task. The second is energy consumption [37,47,48], which is the sum of energy required in completing all tasks. The third is the task completion rate (*CR*), which is the number of tasks successfully completed within the maximum tolerance time divided by the total tasks, and can be expressed by the following formula:

(26) $$CR=\frac{Num_{\mathrm{complete}}}{Num_{total}}$$

Next, we compare five algorithms under three performance indicators. The number of fog nodes is set to 5, 10 and 20, and the task amount is set to 10, 20, 30, 40, 50, 60, 70, 80, 90, and 100.

#### 5.2.1. Maximum Completion Time

**Figure 6:**
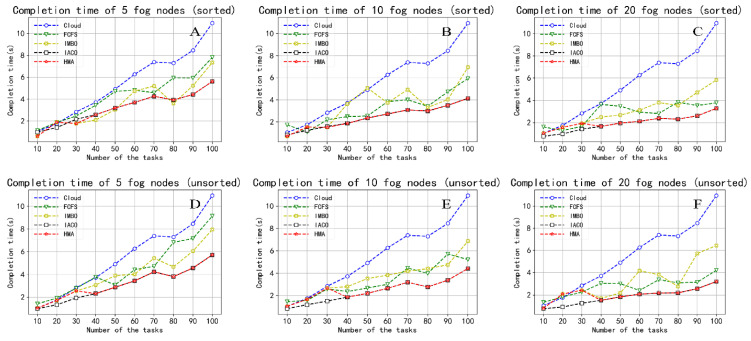
Comparison of the maximum completion time. (**A**) Completion time of 5 fog nodes (sorted); (**B**) Completion time of 10 fog nodes (sorted); (**C**) Completion time of 20 fog nodes (sorted); (**D**) Completion time of 5 fog nodes (unsorted); (**E**) Completion time of 10 fog nodes (unsorted); (**F**) Completion time of 20 fog nodes (unsorted).

For each experiment, before the task scheduling is completed without execution, we set a timer for each cloud/fog node that starts when the first task arrives and stops when the last task is completed. At the end of a task cycle, we compare the times recorded by each node and select the largest one as the maximum completion time for that task scheduling method. Figure 6 illustrates the performance comparison of the maximum completion time between the five task scheduling strategies. Here, Figure 6A–C are the result of sorting tasks, and Figure 6D–F are before sorting. As the number of tasks increases, the task completion time increases. In contrast, as the number of fog nodes increases, it decreases. Furthermore, the cloud has the highest completion time, which may be due to network congestion caused by long-distance transmission between the cloud and the terminal device. While the cloud server has the most robust processing performance, it takes the most time. The remaining algorithms can reduce the huge latency caused by communication by offloading the tasks to the fog nodes. The cloud-fog computing architecture is an effective way to reduce the latency of intelligent production line tasks. When our proposed task rescheduling strategy is used, the latency of IMBO, IACO, and HMA is lower than that of the unused strategy. This shows that the task rushing sequencing strategy based on task priority can reduce the time-sensitive task latency. The proposed HMA can ensure that all tasks can be completed within the maximum tolerance time when the number of tasks is less than or equal to 30 after the tasks are sorted. Furthermore, FCFS scheduling ignores the performance differences between different nodes, resulting in the second-highest delay cost.

#### 5.2.2. Energy Consumption

**Figure 7:**
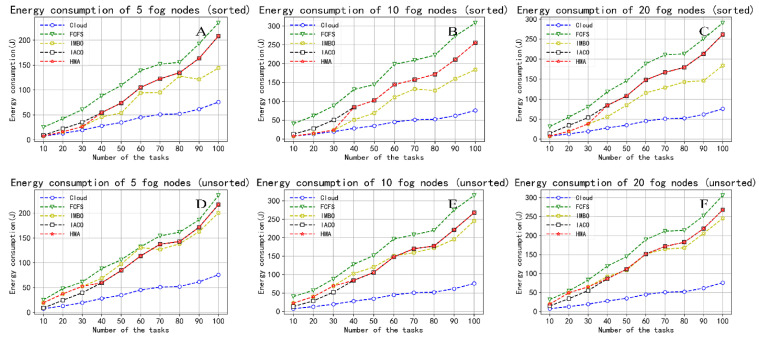
Comparison of the energy consumption. (**A**) Energy consumption of 5 fog nodes (sorted); (**B**) Energy consumption of 10 fog nodes (sorted); (**C**) Energy consumption of 20 fog nodes (sorted); (**D**) Energy consumption of 5 fog nodes (unsorted); (**E**) Energy consumption of 10 fog nodes (unsorted); (**F**) Energy consumption of 20 fog nodes (unsorted).

During the experiment, when the task starts to execute, we calculate the energy consumption required to process each task according to Equation (12). After the task is completed, we use Equation (13) to obtain the energy consumed by all tasks to complete in one task cycle. Figure 7 compares the energy consumption performance between the five task scheduling strategies. Similarly, Figure 7A–C are the result of sorting tasks, and Figure 7D–F are before sorting. We can clearly understand that energy consumption increases with the number of tasks and fog nodes. Here, only the running power consumption of cloud processing tasks is calculated, and the static power consumption of the server is ignored, so the energy consumption brought by cloud services is the lowest. Generally, energy consumption is related to power and running time and decreases as the completion time shortens. The energy consumption of Figure 7A–C is much lower than that of Figure 7D–F, which shows that our proposed task reordering strategy reduces the overall execution time of tasks. It is also worth noting that the energy consumption of HMA is significantly lower than that of FCFS. Traditional scheduling strategies such as FCFS do not consider the load of the fog nodes in the task allocation process, resulting in unbalanced load distribution and higher energy consumption. Equation (14) enables the HMA to adaptively adjust the search direction according to the priority of the task, thus reducing energy consumption. The energy consumption of HMA is less than that of IACO but more than that of IMBO. This shows that in large-scale search problems, HMA has more vital searchability than IMBO. The results obtained better balance time constraints and power consumption, resulting in a slight increase in energy consumption. We believe this is acceptable.

#### 5.2.3. Task Completion Rate

**Figure 8:**
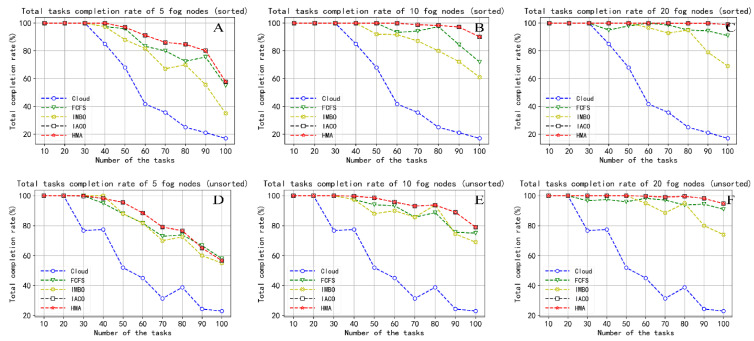
Comparison of the total task completion rate. (**A**) Total tasks completion rate of 5 fog nodes (sorted); (**B**) Total tasks completion rate of 10 fog nodes (sorted); (**C**) Total tasks completion rate of 20 fog nodes (sorted); (**D**) Total tasks completion rate of 5 fog nodes (unsorted); (**E**) Total tasks completion rate of 10 fog nodes (unsorted); (**F**) Total tasks completion rate of 20 fog nodes (unsorted).

In our experiments, we set a timer for each task to keep track of the time it takes from being assigned to completion. We set a counter that counts the number of tasks that can be completed within MaxTime after all tasks are completed. If the task completion time is less than MaxTime, then the counter is incremented by one. MaxTime is different for tasks with different priorities, which are listed in Table 1. Finally, we calculate the task completion rate using Equation (26). Figure 8 shows the completion rate of all tasks within the task tolerance time. We still use Figure 8A–C to indicate the performance results after sorting the tasks, and Figure 8D–F to indicate the results before sorting. According to Equation (26), we know that the trend of the task completion rate is consistent with the maximum completion time. In other words, as the number of tasks increases, the completion rate decreases again, and as the number of fog nodes increases, the task completion rate increases. Whether the task reschedule strategy is adopted, the task completion rate of HMA is always the highest. This is because HMA balances time and task delay through task priority when processing task scheduling, ensuring the priority execution of time-sensitive tasks. When the number of tasks exceeds 30, the completion rate of the IMBO strategy is ranked behind FCFS. As the solution set space increases, the IMBO algorithm is prone to poor performance caused by falling into suboptimal solutions. IACO is the same as HMA in terms of completion rate. Cloud computing is ranked last due to large latency, which results in a low completion rate.

**Figure 9:**
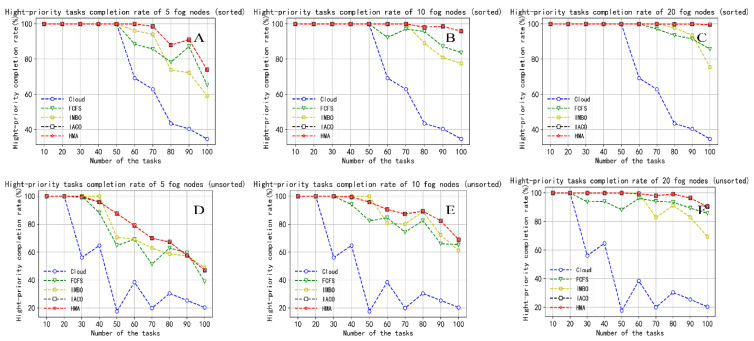
Comparison of the high-priority task completion rates. (**A**) Hight-priority tasks completion rate of 5 fog nodes (sorted); (**B**) Hight-priority tasks completion rate of 10 fog nodes (sorted); (**C**) Hight-priority tasks completion rate of 20 fog nodes (sorted); (**D**) Hight-priority tasks completion rate of 5 fog nodes (unsorted); (**E**) Hight-priority tasks completion rate of 10 fog nodes (unsorted); (**F**) Hight-priority tasks completion rate of 20 fog nodes (unsorted).

We compared the completion rates of high-priority tasks to show the responsiveness of different algorithms to urgent tasks, as shown in Figure 9. Comparing Figure 9A–C and Figure 9D–F, we find that the task rescheduling strategy can improve the completion rate of high-priority tasks, which ensures the requirements of the intelligent production line for time-sensitive tasks. From the information of Figure 9A–C, we can conclude that when the number of tasks is less than 60, the HMA algorithm ensures that all high-priority tasks are completed within the fault tolerance time. This verifies that the task priority-based strategy takes time delay and energy consumption as the optimization goal and converges to the optimal solution better than other algorithms. When the number of fog nodes is 20, all high-priority tasks within 100 are completed. For large-scale tasks, adding a certain number of service nodes can effectively improve the task success rate. The task completion rate of nodes 5 and 10 proves that HMA is superior under the limitation of the number of service nodes. Similar to the total completion rate, IACO ranks second, FCFS leads IMBO when the number of tasks is large, and cloud always ranks last.

In this paper, the proposed HMA scheduling strategy reduces energy consumption as much as possible by reducing the task delay and increasing the completion rate. The experimental results in terms of completion time, energy consumption, and task completion rate show the same trend, proving the feasibility of the proposed strategy in task scheduling. In summary, the experimental results show that the proposed cloud-fog computing architecture and the HMA algorithm based on task priority can provide a rapid response in the intelligent production line. The energy consumption of the intelligent production line system was also reduced.

## 6. Conclusions

This paper highlights the task scheduling problem in intelligent production lines. For the requirement of ultra-low latency, we establish a mathematical model for intelligent production line task scheduling to achieve ultra-low latency and low power consumption of time-sensitive tasks. We transform it into a multi-objective optimization of time delay and energy consumption. Combining the advantages of cloud computing and fog computing, we propose a cloud-fog computing architecture for intelligent production lines and develop a priority-based task rescheduling strategy to ensure that time-sensitive tasks are prioritized services. In addition, we propose the HMA algorithm with a mixture of IMBO and IACO algorithms to solve the optimization problem. This is the first time that the MBO algorithm is used to solve the task scheduling problem of intelligent production lines. We evaluate the performance of HMA in a simulation environment. We find that as the number of tasks increases, the performance improvement also increases. When the number of tasks is 100 and the number of nodes is 10, the maximum completion time is only 37.8% of cloud, 59.6% of IMBO, and 69.9% of FCFS, while the power consumption is 82.9% of FCFS, and the task completion rate is 5.3 times better than cloud, 1.5 times better than IMBO, and 1.25 times better than FCFS. The experimental results show that our proposed strategy can respond quickly to tasks and reduce energy consumption. In the future, we will enhance the proposed task scheduling strategy in the cloud-fog computing architecture to solve the task flow scheduling problem of intelligent production lines.

## Figures and Tables

**Table 1:** Simulation parameters.

Symbol	Value	Description
K	[10,100]	Number of the tasks.
${\mathrm{Cloud}}_{N}$	1	Number of cloud nodes.
${\mathrm{Fog}}_{N}$	[5,10,20]	Number of fog nodes.
${\mathrm{Terminal}}_{N}$	10	Number of terminal services.
$D_{in}$	[1,1024] Kb	Amount of data input for each task.
$D_{out}$	[1,50] Kb	Amount of data output by each task.
Y	[1,10] MIPS	Load of each task.
$C_{cloud}$	204,800 MIPS	Processing rate of the cloud node.
$C_{fog}$	[800,15000] MIPS	Processing rate of fog nodes.
$P_{cloud}$	800 W	Energy consumption of cloud node.
$P_{fog}$	[20,40] W	Energy consumption of fog nodes.
$P_{t}$	[3,5] W	Transmission energy consumption of nodes.
$Dis_{cloud}$	3000 m	Distance between the cloud and other fog nodes.
$Dis_{fog}$	[10,100] m	Distance between the fog nodes.
$MaxTime_{1}$	2 s	Maximum tolerable time for high-priority tasks.
$MaxTime_{2}$	4 s	Maximum tolerable time for low-priority tasks.
ϑ	5.0	Latency weight coefficient.
μ	1.0	Energy consumption weight coefficient.
λ	[1,2]	Task priority.

**Table 2:** Parameters set in Algorithm 1 and Algorithm 2.

Symbol	Value	Description
M	30	Monarch butterfly population size.
$T_{Mob}$	50	Algorithm 1 maximum generation.
p	5/12	Migration ratio.
peri	1.2	Migration period.
BAR	5/12	Butterfly adjusting rate.
$S_{max}$	1	Max walk step.
$N_{ant}$	30	Ant population size.
${Τ}_{ACO}$	300	Algorithm 2 maximum generation.
α	1	Pheromone weight coefficient.
β	5	Weight factor of heuristic information.
ρ	0.5	Pheromone volatilization rate.
$\varphi_{0}$	1.0	Initial pheromone concentration.

## Data Availability

The data presented in this study are available on request from the corresponding author. They are restricted to experimental results.
